# Expanding considerations for treating avoidant/restrictive food intake disorder at a higher level of care

**DOI:** 10.1186/s40337-024-00972-7

**Published:** 2024-01-22

**Authors:** Brianne N. Richson, Danielle C. Deville, Christina E. Wierenga, Walter H. Kaye, Ana L. Ramirez

**Affiliations:** 1 Department of Psychiatry, University of California San Diego Eating Disorders Center for Treatment and Research, 4510 Executive Drive #315, San Diego, CA 92121 USA; 2 Sanford Center for Biobehavioral Research, 120 8th Street S, Fargo, ND 58103 USA; 3 Department of Psychiatry and Behavioral Science, University of North Dakota School of Medicine and Health Sciences, 1919 Elm St N, Fargo, ND 58102 USA; 4 Eating Disorders Center, Children’s Mercy Kansas City, 5520 College Boulevard, Overland Park, KS 66211 USA

**Keywords:** ARFID, Eating disorders, Cognitive-behavioral therapy, Family-based treatment, Neurodevelopment, Executive functioning, Dialectical behavior therapy

## Abstract

Existing descriptions of the treatment of avoidant/restrictive food intake disorder (ARFID) at higher levels of care (HLOC) for eating disorders are limited, despite HLOC settings frequently serving patients with ARFID. The purpose of this commentary is to expand on the preliminary literature that describes pediatric ARFID treatment at HLOC by describing two specific components of our approach to treating pediatric ARFID that may not yet have traction in the current literature. Specifically, we highlight the utility of (1) treatment accommodations that appropriately account for patients’ neurodevelopmental needs (e.g., executive functioning, sensory processing) and (2) the adjunctive use of Dialectical Behavior Therapy (DBT) elements within family-based pediatric ARFID treatment. We also describe necessary future directions for research in these domains to clarify if incorporating these considerations and approaches into pediatric ARFID treatment at HLOC does indeed improve treatment outcomes.

## Introduction

The application of a cognitive-behavioral therapy (CBT), family-based framework to avoidant/restrictive food intake disorder (ARFID) treatment in a higher level of care (HLOC) setting for eating disorders (EDs) has been described [1], as have preliminary ARFID outcomes in a HLOC context [2–5]. This initial evidence base draws from outpatient ARFID treatment approaches [6]. Nevertheless, it is common for patients with ARFID to compose a non-negligible proportion of patients treated at ED HLOC settings, especially child settings [7]. Consensus is lacking on the best approach to treatment for patients with ARFID, particularly within HLOC settings. Patients with ARFID bring unique characteristics affecting treatment objectives, trajectory, and outcome, including earlier onset of symptoms, increased chronicity, neurodevelopmental considerations, frequent medical comorbidities, and increased somatic symptoms [8–11].

In this commentary, we expand on descriptions of existing approaches to treating ARFID in a pediatric population at the partial hospitalization LOC by outlining potentially unique aspects of the treatment we provide and offering associated research directions. Our approach includes CBT for ARFID, family-based treatment, dietary and medical management, therapeutic meals/snacks, group therapy, and parent management training as described in previous studies and case series [1, 5, 12]. Here, we detail two additional aspects of our team’s approach to pediatric ARFID treatment that, to our knowledge, have not been described in the ARFID literature: (1) our efforts to tailor treatment in accordance with patients’ neurodevelopmental presentations, and (2) our integration of Dialectical Behavior Therapy elements (DBT) [13, 14] within the cognitive-behavioral, family-based treatment modalities used with patients and their families.

## ARFID and neurodevelopmental presentation

Relative to other eating disorders, ARFID more frequently co-occurs with differences in neurodevelopment [15, 16], including autism [17–19], attention-deficit/hyperactivity disorder [11, 20], and intellectual developmental disorder [21]. For example, studies in clinical ARFID samples have identified prevalence rates of up to 23.1% for autism [22], up to 40% for attention-deficit/hyperactivity disorder [20], up to nearly 31% for learning difficulties [22], and up to 26% for general cognitive impairment [7], with some variability by ARFID maintaining mechanism. This notable prevalence of various neurodevelopmental diagnoses/differences among patients with ARFID maps onto our clinical observations at HLOC. Moreover, recent longitudinal research indicates that various early neurodevelopmental differences (e.g., differences in attention/concentration, social communication, etc.) associated with neurodevelopmental diagnoses robustly increase the risk for a later ARFID diagnosis [16]. Treatment of patients with ARFID and neurodevelopmental differences presents clinicians with two critical goals that, if not carefully attended to, may be difficult to simultaneously achieve: (1) providing care that is accessible and affirming of neurodiverse presentations, and (2) ensuring that patients are able to achieve medical stability, meet nutritional needs, and reach and/or maintain a healthy weight. Accommodations for patients’ neurodevelopmental presentations are closely considered when designing treatment plans, as described below.

The above neurodevelopmental diagnoses and differences are often characterized by observed divergence in executive functioning (EF) [23–26]. As such, one important neurodevelopmental aspect that informs treatment planning is EF. EF enables engagement in goal-directed cognitive and behavioral regulation [27]. Frequently mentioned manifestations of EF differences include reduced inhibitory control, working memory, and set-shifting abilities [28]. Research on EF in ARFID (in comparison to anorexia nervosa) is extremely limited, although one study identified rigidity (as opposed to flexibility, an aspect of EF) as a significant predictor of selective eating in a range of pediatric sub-samples [29]. One recent study that examined neuropsychological task performance in children and adolescents with ARFID versus AN identified task-dependent deficits in set shifting and cognitive flexibility only in those with ARFID [30]. Whereas these EF-related difficulties are more consistently documented in adults with AN, findings in younger patients with AN appear to be more mixed [31, 32]. Our clinical observation is that many children with ARFID benefit from treatment accommodations to set them up for success in the context of EF difficulties.

Pediatric patients with ARFID and EF difficulties may present with different degrees of abilities to direct attention, to organize and integrate information, to demonstrate flexibility, to engage in planning, to regulate emotions, and to engage in self-monitoring. As a result, certain ARFID patients may present with difficulty shifting from one task or setting to another (e.g., from a difficult meal or snack to a therapy group or vice versa), difficulty tracking time (e.g., during meals), and difficulty with multitasking or completing complex sequential tasks (e.g., completing a self-monitoring log while eating or determining where to start on a multi-component meal, particularly when anxiety is high). Weak central coherence (i.e., preferential focus on details and difficulty integrating details into a ‘whole’ [33) in patients with EF difficulties can make approaching a meal more challenging when one particular component is a feared or non-preferred food. Additionally, certain patients may become overwhelmed by the amount of food presented if presented all at once (resulting in difficulty getting started with eating), and/or have significant difficulty adjusting to meal-plan changes.

For patients who have difficulty approaching a meal due to overwhelm in the presence of a non-preferred food, making accommodations to *what* foods are presented as part of the meal is often useful, with small portions of non-preferred foods perhaps presented alongside their standard meal as a ‘taste test’ that does not factor into nutrition completion at that sitting. This approach is similar to CBT for ARFID wherein non-preferred foods are taste tested in small amounts before the food is incorporated in meal- or snack-sized portions [34]. This may be particularly useful in the treatment of children with longstanding picky eating and sensory sensitivities. In practice, this may involve presentation of meals that meet patients’ caloric needs but are low in variety or do not fit within standard social conventions (e.g., snack foods presented as the primary component at meals with smaller, bite-sized portions of non-preferred foods, nutritional supplements plated alongside meals). Like in CBT for ARFID, this approach prioritizes meeting nutritional needs and creates smaller and more approachable goals to support the introduction of new foods. For children with longstanding eating difficulties exacerbated by underlying sensory differences, it should be noted that the goal of treatment is not to *change* underlying sensitivity but to increase ability to meet nutritional needs in the context of said sensitivities. This approach is similar to the Feeling and Body Investigators (FBI) [35, 36] approach, for example, in which interoceptive sensitivity is harnessed to promote more adaptive engagement with bodily sensations (including acceptance and interoceptive exposures).

For patients who have difficulty initiating eating due to the amount (i.e., volume) of food presented, accommodations may be made to facilitate breaking down the task at hand in a more manageable way, such that patients are presented with, for example, half of their food to start and the remaining half upon completion of the first half. Patients with self-monitoring and/or time-tracking difficulties may benefit from more frequent reminders and prompts regarding the amount of time remaining in the meal to facilitate appropriate pacing. We also aim to make self-monitoring tools as accessible as possible by keeping time-tracking tools such as sand timers, simple thought records/exposure logs, and visual reminders of patients’ reward systems in the meal room.

In addition to food-related sensory sensitivity that is associated with ARFID, we often observe differences in broader sensory needs. For example, due to both EF factors described above and sensory sensitivity, patients may require a separate seating area or a strategic seating arrangement if the meal room or certain parts of the meal room can become overwhelming from a sensory perspective due to noise, smell, or other distractions. It is also common for patients’ treatment plans to accommodate extra distraction tools during meals and snacks given sensory needs (e.g., headphones, a tablet, drawing materials, inflated balance cushions or fidget kick bands on chairs to assist with the need for extra movement). In these scenarios, we aim to provide gradual scaffolding to help patients acclimate to an environment that may be more akin to what their outside-of-treatment eating environments may look like (e.g., school lunchrooms or other settings in which accommodations may not be possible). Notable sensory needs may also present in patients’ engagement in milieu groups. Patients may require more frequent breaks from groups or seats that help reduce distraction and accommodate movement needs.

To work within the context of patients’ existing EF, the previously described strategies all aim to modify the patient’s environment to meet treatment goals and maximize functioning. For patients with unique neurodevelopmental considerations and/or lifelong issues related to sensory sensitivity, mealtime accommodations may need to be incorporated into daily living following discharge from eating disorder treatment to make the goal of meeting nutritional needs more accessible, in line with the goal of providing care that is affirming of neurodiversity and sets children up for success. This may differ from short-term strategies typically used to assist with mealtime difficulties during eating disorder treatment among children who are otherwise neurotypical and aim to return to prior eating patterns. As described below, additional strategies that focus on skill development (rather than environmental modification) are also typically applied in tandem to influence behavior change and ultimate outcomes.

## Applying a DBT framework to pediatric ARFID treatment

It is not uncommon for DBT to be used in HLOC ED treatment for both youth and adults due to DBT’s emphasis on emotion regulation and distress tolerance skills [37, 38]. The use of distress tolerance skills in HLOC ARFID treatment specifically has also been briefly described [1]. Although there is not an evidence base for or against the use of DBT in the context of ARFID treatment, our clinical observations suggest that there are aspects of DBT that complement a cognitive-behavioral, family-based ARFID treatment framework, as described below. In particular, we focus on the role of DBT in parents’ and caregivers’ treatment experiences.

In our treatment approach, parents receive weekly didactic instruction in DBT skills. Pediatric ARFID patients also learn developmentally tailored versions of the same DBT skills in a separate weekly group, alongside patients who present with other eating disorder diagnoses. Although we consider cognitive-behavioral and exposure-based approaches the core components of patients’ individual therapy treatment plans, patients are encouraged to use DBT skills when appropriate (e.g., to help facilitate nutrition completion, to increase willingness to engage in an exposure, etc.). These parallel groups facilitate the development of an additional shared framework and unified language pertaining to skills, emotions, and behaviors between parents and patients. Parents also benefit from receiving validation and problem solving through interactions with other parents and caregivers in this group setting.

Through DBT skills groups, parents learn mindfulness, distress tolerance, emotion regulation, and interpersonal effectiveness skills. Parents are taught to apply these skills in their own lives, particularly when interacting with their children. As an example, a parent may learn to apply mindfulness practices as taught in DBT to non-judgmentally observe moments of dysregulation or distress in their child, notice and attend to aspects of their own emotional responses, and be intentional about engaging an effective response. They may also encourage the use of mindfulness strategies (e.g., observing, describing) when guiding their children to approach new foods, which is consistent with guidance encouraging patients to approach novel foods nonjudgmentally in CBT for ARFID [34]. Given that meals are frequently distressing for children with ARFID and their parents, parents are encouraged to apply emotion regulation and distress tolerance skills to manage their own vulnerability factors, learn to attend to their emotional experiences, and to tolerate and/or effectively modulate emotional responses that arise during refeeding. These skills offer a resource to parents who are engaging children in distressing aspects of treatment, such as exposures or navigating gastrointestinal discomfort associated with refeeding. Finally, parents also learn interpersonal effectiveness skills, which emphasize the importance of interpersonal validation. Many of the patients with ARFID presenting to our HLOC report difficulties with eating and anxiety that have been longstanding, if not lifelong. It can be challenging for parents to appreciate how challenging the task of eating is for their children, which can result in inadvertent invalidation at mealtimes. Interpersonal effectiveness skills help parents validate how difficult ARFID treatment is for their child while simultaneously supporting the approach of discomfort and/or confrontation of fearful situations.

Also drawing from a DBT framework, parents receive access to 24/7 phone coaching delivered by their family therapist. While patients undergo treatment, parents are responsible for plating meals and snacks outside of program hours. Phone coaching serves to provide in vivo support to empower parents to make effective real-time decisions regarding their child’s eating and behavior. This creates opportunity for parents to build mastery around the skills introduced in treatment and gain support during challenging meals that they may not have access to in less intensive care.

## Research directions

The above-described elements of our approach to treating ARFID in a pediatric HLOC context suggest several future research directions. There is a need for research on the ways in which diversity in neurodevelopmental presentations impacts pediatric ARFID treatment outcomes, and the ways in which accommodations of treatment with respect to neurodevelopmental differences may improve outcomes. Future research in this population should consider the inclusion of EF measures in treatment assessment batteries. Although in this commentary we have described adaptations to treatment given patients’ EF and other neurodevelopmental considerations, cognitive interventions intended to improve EF in the context of EDs do exist [39]. However, the existing EF/ED literature has largely focused on EDs other than ARFID. Similarly, a larger body of research exists on adapting ED treatment for autistic patients with anorexia nervosa [40, 41] given these patients’ often different sensory and cognitive needs. Future research should consider if treatment adaptations related to both EF and sensory needs can similarly benefit patients with ARFID.

Future research in HLOC ED settings that use DBT is needed to examine the efficacy of DBT as an adjunct to core ARFID treatment. More foundationally, additional studies clarifying the extent to which DBT targets (e.g., emotion dysregulation) play a role in ARFID pathology and outcomes are needed. Thus, like our recommendations for assessing the impact of EF on outcomes, it would be useful for emotion dysregulation assessment to become more standard in ARFID treatment outcome batteries. Additionally, studies could examine whether parent DBT skill use predicts treatment outcomes in children and adolescents with ARFID, similar to existing research on timing of DBT skill uptake in adults with EDs at HLOC [42]. It will also be important for future research to clarify whether parent phone coaching and/or parent skill use influence treatment outcomes by, for example, impacting parental self-efficacy. Increased parental self-efficacy contributes to favorable family-based treatment outcomes in outpatient treatment of anorexia nervosa [43] and has also demonstrated preliminary evidence for correlations with favorable ARFID treatment outcomes [44]. Moreover, there is evidence to suggest that partial hospitalization settings do not *decrease* parental self-efficacy despite patients spending less time at home with families during refeeding [45, 46]. On the contrary, this provides further support for incorporating parent interventions at HLOC as this can better equip parents to continue treatment progress in outpatient LOC and aid in the successful application of these interventions in a patient’s natural environment.

## Conclusions

Our clinical observations suggest that ARFID treatment in a pediatric HLOC setting is augmented by attending to patients’ neurodevelopmental presentation and promoting parents’ use of DBT skills, despite little research on these treatment components in ARFID. Particularly at HLOC, where patients with ARFID may not have responded to outpatient care, patients’ neurodevelopmental differences and parents’ own psychological skillset may be highly relevant to treatment engagement and, consequently, outcomes.

## Data Availability

Not applicable.

## Abbreviations

ARFID: Avoidant/restrictive food intake disorder
CBT: Cognitive-behavioral therapy
DBT: Dialectical behavior therapy
ED: Eating disorder
HLOC: Higher level of care
